# Bispecific antibodies and CAR-T cells: dueling immunotherapies for large B-cell lymphomas

**DOI:** 10.1038/s41408-024-00997-w

**Published:** 2024-02-08

**Authors:** Asaad Trabolsi, Artavazd Arumov, Jonathan H. Schatz

**Affiliations:** 1 Sylvester Comprehensive Cancer Center, University of Miami Miller School of Medicine, Miami, Fl USA; 2 Hematology-Oncology Fellowship Program, Jackson Memorial Health System/ University of Miami, Miami, Fl USA; 3 Division of Hematology, Department of Medicine, University of Miami Miller School of Medicine, Miami, Fl USA

**Keywords:** B-cell lymphoma, Immunotherapy

## Abstract

Despite recent advances in frontline therapy for diffuse large B-cell lymphoma (DLBCL), at least a third of those diagnosed still will require second or further lines for relapsed or refractory (rel/ref) disease. A small minority of these can be cured with standard chemoimmunotherapy/stem-cell transplant salvage approaches. CD19-directed chimeric antigen receptor T-cell (CAR-19) therapies are increasingly altering the prognostic landscape for rel/ref patients with DLBCL and related aggressive B-cell non-Hodgkin lymphomas. Long-term follow up data show ongoing disease-free outcomes consistent with cure in 30–40% after CAR-19, including high-risk patients primary refractory to or relapsing within 1 year of frontline treatment. This has made CAR-19 a preferred option for these difficult-to-treat populations. Widespread adoption, however, remains challenged by logistical and patient-related hurdles, including a requirement for certified tertiary care centers concentrated in urban centers, production times of at least 3–4 weeks, and high per-patients costs similar to allogeneic bone-marrow transplantation. Bispecific antibodies (BsAbs) are molecular biotherapies designed to bind and activate effector T-cells and drive them to B-cell antigens, leading to a similar cellular-dependent cytotoxicity as CAR-19. May and June of 2023 saw initial approvals of next-generation BsAbs glofitamab and epcoritamab in DLBCL as third or higher-line therapy, or for patients ineligible for CAR-19. BsAbs have similar spectrum but generally reduced severity of immune related side effects as CAR-19 and can be administered in community settings without need to manufacture patient-specific cellular products. To date and in contrast to CAR-19, however, there is no convincing evidence of cure after BsAbs monotherapy, though follow up is limited. The role of BsAbs in DLBCL treatment is rapidly evolving with trials investigating use in both relapsed and frontline curative-intent combinations. The future of DLBCL treatment is bound increasingly to include effector cell mediated immunotherapies, but further optimization of both cellular and BsAb approaches is needed.

## Introduction

Diffuse large B-cell lymphoma (DLBCL) is the most common non-Hodgkin lymphoma, with nearly 60,000 expected new diagnoses in the United States and Western Europe during 2023. Incidence is rising annually along with average population age, projected to increase from 29,108 new U.S. cases in 2020 to 32,443 in 2025 [1]. Despite recent improvements in treatment options, 30–40% of those diagnosed still ultimately will die from complications of the disease [2]. Advances in frontline therapy have distinct milestones. Addition of rituximab to CHOP (cyclophosphamide, doxorubicin, vincristine and prednisone) improved overall survival from 57% to 70% in the pivotal trial by Coiffier et al. in 2002 and represented to first improvement over CHOP [3, 4]. Many attempts to add agents or intensify dosing or frequency of chemotherapy did not improve outcomes, and only targeting the immunophenotype of DLBCL via CD20 with rituximab, or two decades later via CD79B with the antibody drug conjugate polatuzumab vedotin (polatuzumab), led to front-line therapeutic leaps. The randomized POLARIX trial replaced vincristine with polatuzumab, creating Pola-R-CHP, which won approval as a frontline treatment option in the United States and Europe based on improved progression-free survival (PFS) compared to R-CHOP [5]. Though many clinicians may choose Pola-R-CHP for cases with inferior-prognosis activated B-cell (ABC) cell-of-origin (COO) phenotype, which seemed to drive results in subgroup-analyses, POLARIX was not powered for such conclusions, and in addition, an overall survival (OS) benefit of Pola-R-CHP is not yet established. A recent meta-analysis provided further support for improved activity of polatuzumab specifically for ABC cases, including those identified through routine immunohistochemical staining rather than gene-expression profiling [6].

Cancer immunotherapy is a broad term that includes naked antibodies, bispecific antibodies (BsAbs), antibody-drug conjugates, immune-checkpoint inhibitors (ICIs), chimeric antigen receptor T cell therapy (CAR-T), cancer vaccines, and allogeneic stem-cell transplantation. It was first tested more than a century ago. William Coley – known now as the father of immunotherapy – noticed spontaneous cancer regression in patients who had simultaneous bacterial infections. He reported clinical use of bacterial injections to induce regression of “lympho-sarcoma” [7], work that was not widely acknowledged for decades. Advances in immunology and cancer research accelerated with the discovery of interferon in 1957 [8], characterization of T-cells, dendritic, and natural killer cells in the 1970s [9–11], and accumulating knowledge on bone marrow transplantation pioneered at the University of Minnesota in the 1980s [12]. The latter established high-dose therapy with autologous stem cell rescue (HDT/ASCR) as consolidative treatment of choice for relapsed lymphoma [13], with allogeneic stem-cell transplantation (SCT) reserved for selected cases due to higher rates of treatment-related morbidity and mortality. Allogeneic SCT utilizes a donor’s immune system including, innate and adaptive components, to both replenish the bone marrow and target cancer with graft-versus-tumor effect. This “shotgun” approach however distinguishes poorly components of the immune system needed for anti-tumor activity vs. those that only add morbidity, in particular graft-versus-host disease, a common and potentially fatal complication.

T-cells have been widely explored in therapeutic approaches for cancer. Rosenberg and colleagues described therapeutic infusions of lymphokine activated killer cells and later, tumor infiltrating lymphocytes (TILs), ushering in the era of autologous immunotherapy [14]. With modest but clear responses in immunogenic tumor types like renal cell carcinomas and melanomas, TILs established proof of principle for T-cell therapy and propelled innovative approaches to harness its power. It was the introduction of ICIs and CAR-reprogrammed T cells, however, that truly moved the needle and placed T-mediated immunotherapies among the pillars of cancer treatment [15–17]. The CAR-T concept was first described by Eshhar et al. in 1992 in Israel when they constructed chimeric genes composed of a single-chain variable fragment domain (scFv) of an antibody linked with gamma or zeta chains, the common signaling domains of the immunoglobulin receptor and the T cell receptor (TCR), thus endowing T cells with antibody-mediated antigen specificity [18]. To enhance function and avoid apoptosis, co-stimulatory domains were added (CD28 or 41BB), creating second-generation CARs that are the basis of currently approved CAR-T therapies [19]. Autologous CAR-T cells were first administered in humans in the mid-late 2000s [20] followed by multiple publications of its early use in humans [21, 22], and is today approved for B-lymphoid malignancies including acute lymphoblastic leukemia (ALL), indolent and aggressive lymphomas [23–27], and multiple myeloma [28–30]. It requires, however, specialized centers to harvest, engineer and reinfuse these products, a process that takes 20–40 days and hampered by occasional failure. Many strategies are under development to overcome these shortcomings, including development of allogeneic “off-the-shelf” CAR-T cells not requiring manufacture on a per-patient basis. BsAbs are meant to be easier still – a single compound that can be infused to both activate patients’ T cells and target them to tumors. BsAbs comprise distinct binding sites for at least two specific antigens, one on target tumor cells, the other on effector T-cells (typically CD3) thus inducing a cytotoxic cellular response [31].

In this review we explore the clinical development and use of BsAbs in DLBCL from early reports of blinatumomab activity and its shortcomings to the development novel efficacious next-generation constructs now entering clinical practice. We will contrast BsAbs efficacy with the curative potential of CAR-19, a feat yet to be proven for BsAbs. We also explore combinations and sequencing of BsAbs with CAR-T while providing thoughts into the future of DLBCL immunotherapy and whether BsAbs will replace CAR-T cells, enhance their function, or merely provide an alternative.

## CAR-19 in DLCBL

While advances in bispecific antibody treatment of DLBCL are exciting, it’s necessary to evaluate their results in the context of CAR-19, where longer follow up allows clearer conclusions. Three CAR-19 products – Axicabtagene ciloleucel (axi-cel), lisocabtagene maraleucel (liso-cel), and tisagenlecleucel (tisa-cel) – are approved for rel/ref LBCL patients after three or more prior lines (Table 1). In addition, Axi-cel and liso-cel demonstrated superior progression-free survival compared to standard salvage chemoimmunotherapy followed by HDT/ASCT for second-line LBCL patients either refractory to frontline or relapsed within 12 months, and are approved for use in this setting [27, 23, 24, 32]. Indeed, updated long-term follow up of ZUMA-7 after median 47.2 months showed median OS was not reached for axi-cel compared with 31.1 months for the standard of care [33]. Real-world data from the U.S. Car-T Consortium and the French DESCAR-T registry, focusing primarily LBCL patients receiving CAR-19 as third or later line therapy, are consistent with long-term disease free survival of about in in such patients [34, 35]. These long-term data, rare PFS events beyond two years, and clear tail on OS curves illustrate the curative potential of CAR-19.

**Table 1:** Clinical characteristics, outcomes and toxicities of CAR-T Phase III trials in large B cell lymphoma (LBCL).

	Axi-cel	Tisa-cel	Liso-cel
B cell target	CD19	CD19	CD19
Stimulatory Signal 1	CD3ζ	CD3ζ	CD3ζ
Stimulatory Signal 2	CD28	4-1BB	4-1BB
Phase 3 Trial	Zuma-7	Belinda	Transform
Bridging Therapy	Not allowed	83% received bridging; platinum-based	64% received bridging;
Median time from leukapheresis to infusion	29 days	52 days	26 days
ORR	83%	75%	87%
CRR	65%	46%	74%
Median PFS	14.7 months	3 months (EFS)	NR (18 m PFS = 58%)
Median OS	NR	15.3 months	NR (18 m OS = 73%)
Median follow up	47.2 months	10 months	17.5 months
DOR	26.9 months	–	NR
FDA approval in LBCL	2^nd^ line: primary refractory or relapsed in one year LBCL ≥3^rd^ line: relapsed refractory LBCL	2^nd^ line: not approved 3^rd^ line: relapsed refractory LBCL	2^nd^ line: primary refractory or relapsed in one year LBCL. Patients with ≥1 PLT and in eligible for ASCT. ≥3^rd^ line: relapsed refractory LBCL
CRS grade ≥3	6%	5%	1%
Neurologic AEs grade ≥3	21%	2%	4%

*AEs* adverse events, *ASCT* autologous stem-cell transplant, *CRR* complete remission rate, *CRS* cytokine release syndrome, *DOR* duration of response, *EFS* event-free survival, *NR* not reached, *ORR* overall response rate, *OS* overall survival, *PFS* progression-free survival, *PLT* prior lines of therapy.

Shortcomings of CAR-T can be divided into logistical challenges and toxicity related adverse events (AEs). Logistically, access to CAR T-certified centers is restricted by geographic and socioeconomic factors. Disparities are documented with one study reporting that only a third of African Americans lived in a county with a CAR-T or bispecific antibodies trial. Center certification is a lengthy and resource-exhaustive process that can take 6–18 months and is fraught with challenges and redundancies [36]. Analyses show 29–71% of estimated medically eligible rel/ref DLBCL patients, are not treated with a licensed CAR T-cell therapy [17, 37–39]. CAR-19 also contributes to increased hospitalization. From its initial 2017 FDA approval through 2019, immunotherapy admissions for lymphoma underwent 2.7-fold increase in length of stay and 10-fold increase in total charges [40]. By 2019, lymphoma became the most frequent malignancy in patients receiving inpatient immunotherapy. Other than logistical and eligibility constraints, AEs of CAR-19 are notable, and thus guidelines by the European society for Blood and Marrow Transplanation have been developed to aid in managing CAR-19 related toxicities [41]. Cytokine release syndrome (CRS) occurs in 50–90% of patients (6% grade ≥3) while immune effector cell-associated neurotoxicity syndrome (ICANS) grade ≥3 was reported at 4–21% [24, 27, 32]. These numbers are lower for liso-cel, which differs from the other two products by blending equal numbers of CD4 and CD8 CAR-T cells [23]. Toxicity concerns increasingly are mitigated by growing expertise in AE management in CAR-T centers, including prompt administration of the interleukin-6 (IL-6) receptor-blocking antibody tocilizumab. In addition, IL-1 blockade mitigates ICANS in preclinical models [42], and the IL-1 receptor antagonist anakinra, FDA-approved to treat rheumatoid arthritis, has entered clinical investigation for treatment of ICANS Gr ≥2 with promising results in small, uncontrolled phase 2 study [43]. CAR-19 therefore has curative potential in rel/ref DLBCL, but limited access and selection criteria ultimately reduce the number of patients able to benefit from it.

Recent guidance from the FDA in November 2023 noted a potential risk for onset of secondary T-cell malignancies after CAR-T therapy [44], adding a layer of complexity, some would argue unnecessary confusion, to clinical decision making. More than 34 000 patients have been treated with CAR-T therapy to date [45], and the FDA alert is based on reports of approximately 20 T-cell malignancies developing later. It’s well established from the pre-CAR era that patients treated for B-cell lymphomas have risk of developing subsequent T-cell malignancies at a standardized incidence ratio of 4.7 compared to the general population [46]. Sporadic secondary T-cell lymphomas at such a tiny rate therefore likely has no specific link to CAR-T therapy, unless a case is shown to be CAR-positive, i.e. derived from the engineered product itself. There is a single case report of this, derived from the BCMA-targeted myeloma product ciltacabtagene autoleucel (cilta-cel), that was presented as a publication-only abstract at the recent 2023 American Society of Hematology annual meeting [47]. Even here, based on the mutational profile of the tumor, there was no clear role of instertional mutagenesis by the CAR construct in driving onset. Experimental use of the *piggyBac* retrotransposon system to generate CD19 CAR-T cells resulted in 2 of 10 patients developing CAR-positive lymphomas [48], but this system is not used in the manufacture of any FDA-approved CAR product. It should also be noted that other secondary cancers post-CAR-T, particularly myeloid malignancies, are described at what appear to be substantially higher rates than T-cell malignancies, though here again it’s not clear if the increase relates to CAR-T or well established underlying factors of age and prior cytotoxic cancer treatments [49, 50]. Anecdotal reports on social media of patients declining potentially curative CAR-T therapy following the FDA alert shows the importance of careful counseling in the clinic and further clarity from the FDA itself moving forward.

## Development bispecific antibodies in DLBCL

**Fig. 1:**
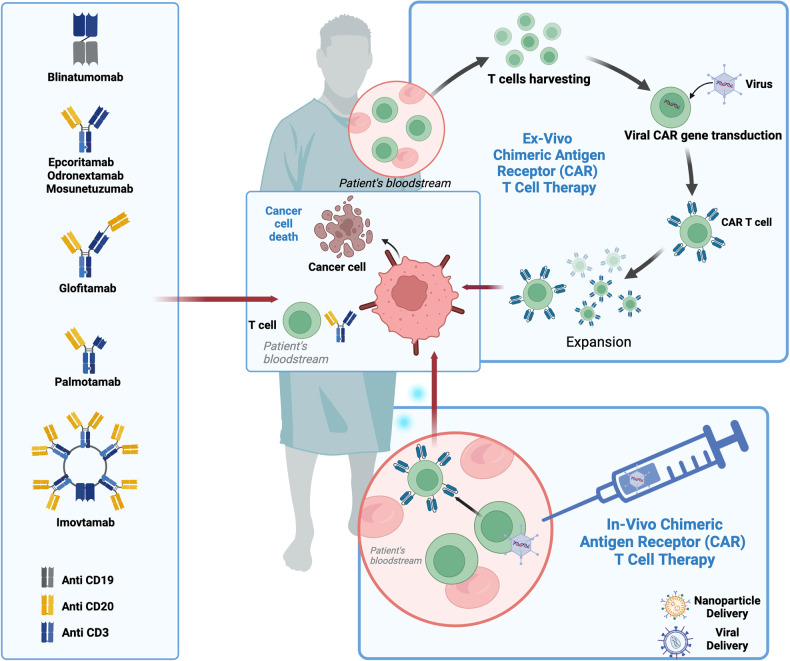
Landscape of effector cellular therapy for DLBCL therapy. Bispecific T cell engagers (left) include BiTEs like blinatumomab, fused full-length antibodies like the DLBCL-approved products epcoritamab and glofitamab, and multivalent constucts like imovtamab. Approved CAR-19 therapies (top right) are manufactured ex vivo from each patient’s T cells, requiring 20–40 days. Viral or nanoparticle delivery of CAR genes (bottom right) in vivo is one of many investigational ways to potentially accelerate targeted cell therapy delivery.

The preclinical journey of BsAbs dates back 30 years to work by German scientists Mack et al., who linked two different scFv fragments through a chemical linker, a design that later came to be called a BiTE or bispecific targeted engager. Their initial lead molecule bound CD3 on human T cells and the epithelial antigen 17-1A, frequently expressed on colorectal cancer [51]. This paved the way for generation of blinatumomab the first clinical BiTE approved in the United States, directing T cells to CD19, a specific B-cell surface protein (Fig. 1). B-cell depletion is tolerable in humans and hence a large portion of early development in BsAbs focused on B lymphomas and leukemias [52]. Subsequent development and clinical trials lead to approval in B-ALL in 2017, making blinatumomab the first bispecific with regulatory approval in cancer [53–55]. The design of two adjoining scFv domains, while compact with a molecular weight of 55kD, confers rapid renal clearance, necessitating continuous infusion [51, 53]. Blinatumomab was investigated in DLBCL and showed overall response rate (ORR) 43% including complete responses (CRs) in 19% [56]. Other Phase 2 studies (NCT02910063) were performed, but, in the context of CAR-19’s approval, and high required doses and concomitant toxicities for efficacy in LBCL, sponsor decision was not to pursue further lymphoma development. Simultaneous with emergence of blinatumomab, other constructs with varying molecular sizes were under development. These include larger molecules that fuse the arms of two distinct full-size antibodies through their respective Fc domains. Attachment of Fab domains in a variety of configurations allows constructs that facilitate 2:1 binding, or large IgM-like pentamers, to name a few [57]. Of these, lead candidates emerged, all of which are IgG full antibody-based (Fig. 1). Glofitamab, epcoritamab, and mosunetuzumab were the first of these second-generation constructs to progress toward approvals for DLBCL [58]. Notably, all target CD20 rather than CD19. CD19 is present from the early pro-B step in maturation through terminal differentiation to plasma cells, while CD20 is acquired in more mature pre-B cells and is lost during plasma-cell differentiation. Horna et al. examined expression differences in B-cell lymphoma biopsies and found overall higher density of CD20 surface expression, while CD19 was more heterogeneous and was preserved in few CD20-negative tumors [59]. Possibly more important than these potential advantages is the loss of CD19 surface density, in some cases complete genomic *CD19* loss, that may be found in cases relapsed or refractory after CAR-19 [60–62]. CD20, on the other hand, is the target of rituximab, the most widely used medication in DLBCL, but is rarely absent from tumors relapsing later. While mosuntetuzumab targets the same epitope as rituximab, epcoritamab and glofitamab target epitopes shared by ofatumumab, a much less used antibody in DLBCL [58, 63].

## Clinical results of bispecifics in DLBCL

Epcoritamab and glofitamab were granted accelerated approval by the FDA in May and June of 2023, respectively, making them the first BsAbs to be approved for DLBCL [64, 65] (Table 2). Epcoritamab was initially tested in phase1/2 dose escalation in rel/ref NHL [66]. ORR was 75% with 69% CRs among evaluable patients receiving full doses. Importantly, no Grade 3 toxicities were reported. The study included 22 DLBCL patients receiving full doses, of whom 15 (68%) achieved response and 10 (45%) had CR at a median follow up of 9.3 months. In dose expansion, 157 patients with rel/ref LBCL received weekly step-up dosing to 48 mg on day 15 in Cycles 1-3 (C1-C3), every 2 weeks (W) (C4-C9), then every 4WC ≥ 10 until progression. Most patients (61%) had disease refractory to prior therapy, including 40% who had received CAR-19. At median follow up 10.7 months, ORR was 63% with 40% CRs. Median duration of response (DoR) was 12 months, and 88.7% of complete responders remained in response at 9 months. In subgroup analysis, patients who were not refractory to prior therapy and who had responded to anti-CD20 therapy had improved CR rate. CAR 19-refractory patients had decreased ORR but similar CR rate, while prior ASCT had no predictive value for either. The large number of patients previously exposed to CAR-19 (38.9%, *n* = 61) in this study demonstrated efficacy of epcoritamab in patients with prior exposure to either CAR-T or ASCT. Epcoritamab also induced remissions in patients with aggressive disease refractory to both first and/or last-line of therapy, a notable point given these populations are the most challenging to successfully bridge and enroll onto CAR-T therapy, providing evidence for the clinical advantage of ready-to-use BsAbs with respect to CAR-T. While traditional adverse prognostic factors such as non-germinal center (non-GCB) phenotype and transformed DLBCL were neutral, primary refractory patients remained the most challenging population [67]. Recently, the EPCORE NHL-01 trial results were updated at the International Conference on Malignant Lymphoma 2023. With median follow-up 20 months, ORR and CRs for DLBCL were 61.9% and 39.6%, respectively. The median duration of CR was 20.8 mo. Median OS was 18.5 months for patients with LBCL overall and 19.4 months for patients with DLBCL specifically [68].

**Table 2:** clinical characteristics and outcomes of select BsAbs in diffuse large B cell lymphoma (DLBCL).

BsAb	Target	Schedule and logistics	Population	Results	Grade ≥3 AEs	FDA indication
Epcoritamab	CD20xCD3	SC, weekly (C1-C3), every 2W (C4–C9), every 4WC ≥ 10. Given till progression Steroid Ppx	≥2 PLT including ASCT and CAR-T	ORR 63.1% (99/157) CR 38.9% (61/157) DOR 12 m	Any 61.1%, AEs leading to termination 7%, CRS 2.5%, ICANS 0.6%, TLS 1.3%	Accelerated approval ≥2 PLT in r/rDLBCL, NOS, T-iNHL and HGBCL.
Mosunetuzumab	CD20xCD3	IV, C1D1: 1 mg; C1D8 2 mg; C1D15 and C2D1: 60 mg; C3 + D1: 30 mg. (fixed duration up to C8 for pts in CR and C17 for pts in PR/SD)	≥2 PLT including ASCT and CAR-T Types: DLBCL, T-FL	ORR 42.0% (37/88) CR 23.9% (21/88) DOR 7 m PFS 3.2 m OS 11.5 m	Any 58%, AEs leading to termination 4.5%, CRS 2.3%, ICANS 0%, grade 5 TRAEs 1.1%	Not yet approved for DLBCL
Glofitamab	CD20xCD3	IV, weekly step up dosing starting on d8 (2.5 mg), d15 (10 mg) of C1, followed by a dose of 30 mg on d1 of C2–12 (fixed duration) Obinutuzumab pretreatment 7d before therapy	≥2 PLT including ASCT and CAR-T Types: DLBCL, HGBCL, PMBCL, T-FL	ORR 51.1% (80/155) CR 39% (60/155) DOR 18.4 m PFS 4.9 m, OS 11.5 m	Any 56%, AEs leading to termination 9%, CRS 3.9%, ICANS 3%, TLS 1%, FN 3%	Accelerated approval ≥2 PLT in r/r DLBCL, NOS or T-FL LBCL
Odronextamab	CD20xCD3	IV, weekly (C1–C4) then every 2 weeks Steroid Ppx	≥2 PLT, including prior CAR-T	ORR 52% (66/127) CR 31% (39/127) Duration of CR 17.9 m	Any 78.6%, AEs leading to termination 10%, CRS 1%, ICANS 0%,	Not yet approved for DLBCL
Palmotamab^a^	CD20xCD3	IV, ≥2 cycles if PR achievd	Median number of prior lines:4, 50% CAR-T exposed	OR 47.4% (9/19 pts) CR 26.3% (5/19 pts)	Any 77.8%, AEs leading to termination 13.9%CRS 0%	Not yet approved for DLBCL

*AEs* adverse events, *CR* complete response, *DOR* duration of response, *FN* febrile neutropenia, *IV* intravenous, *M* months, *ORR* overall response rate, *PMBCL* primary mediastinal B cell lymphoma, *Ppx* prophylaxis, *PR* partial response, *r/r* relapse/ refractory, *SD* stable disease, *SC* subcutaneous, *FL* transformed follicular lymphoma, *T-iNHL* transformed indolent non-Hodgkin lymphoma, *TRAEs* treatment related adverse events, *W* weeks.

^a^Phase 1 dose escalation data.

Glofitamab was designed for higher target-cell affinity with a bivalent CD20 targeting moiety and monovalent anti-CD3 (2:1 design, Fig. 1). In phase 1 dose escalation, 177 patients with rel/ref NHL, 74.3% of whom had DLBCL, achieved a 53% ORR with 36.8% CRs and yielded a recommended phase 2 dose of 30 mg every 21 days [69]. In dose expansion, 155 patients with DLBCL, primary mediastinal B cell lymphoma (PMBCL), high grade B cell lymphoma (HGBCL), and transformed follicular lymphoma (tFL) received glofitamab; 33% had received prior CAR-19. To minimize CRS, one pretreatment dose of obinutuzumab (1000 mg) was added 7 days before initiating step-up glofitamab dosing on D8 and D15 of the first cycle, then D1 of each 21 day cycle thereafter for a prespecified total of 12 cycles. CR occurred in 39% at median follow up of 12.6 months. At 12 months, 78% of complete responders remained in CR. Patients that were non-refractory to most recent therapy and/or non-refractory to ASCT had improved chance of response. Data on post-CAR-19 patients were not provided. COO and double-hit (simultaneous *MYC* with *BCL2* rearrangements) status had no predictive value [70]. A recent update for this trial showed 66% of CRs maintained it at 18 months, median duration of CR of 24.1 months [71]. Approved as monotherapy in the rel/ref setting, glofitamab is also under investigation in combination with polatuzumab. In a phase 1/2 study, 111 patients received this combination. Gr ≥3 AEs occurred in 61% of pts, most commonly neutropenia (30%; one febrile neutropenia event). The most common AE was CRS (44%), mostly Gr 1/2, though one patient suffered Gr 5. Best CR rate of 56% and median DoR was 17.9 months [72]. It remains to be seen how this can be applied in practice and whether a phase 3 trial will confirm these findings.

Other bispecific antibodies are also well into clinical testing and potentially nearing review for FDA approvals. Odronextamab is a CD20xCD3 BsAb evaluated in the ELM-2 phase 2 trial in DLBCL patents after ≥2 prior lines of therapy (PLT). ORR was 52% (66/127) with 31% CRs (39/127) and median DoR 10.2 months. In patients with prior CAR-19 therapy (*n* = 31), CR rate was maintained (32.3%). Notably, 6 patients died of treatment-related AEs, warranting further investigation of mitigation strategies [73, 74].

Mosunetuzumab was assessed in phase 1 study in patients with multiple types of rel/ref B-cell NHL, followed by dose expansion in DLBCL. Eighty-eight patients (73.9% de-novo DLBCL; 26.1% transformed follicular lymphoma) were enrolled. Nearly a quarter (23.9%) achieved CR, and ORR was 42.0%. Median PFS was 3.2 months. The CR rate in 26 patients who had received prior CAR-19 was 12%. CRS occurred in 26.1% of patients and primarily in cycle 1 [75, 76]. Though difficult to compare in non-head-to-head settings, these data suggest lower efficacy of mosunetuzumab, and combinations with other treatments are under evaluation [77, 78]. For example, the phase 3 SUNMO study is a randomized comparison of mosunetuzumab + polatuzumab against R-GemOx (rituximab, gemcitabine, oxaliplatin) in patients with ≥2 PLT (1 if ASCT ineligible). Although a control arm of polatuzumab, bendamustine and rituximab (Pola-BR) would more directly assess mosnetuzumab’s impact compared to chemo, the results of SUNMO nonetheless could help establish a role for the compound in later lines of treatment [79]. Bispecific antibodies at earlier stages of evaluation include palmotamab [80] and imvotamab (igm-2323). The latter construct has 10 high-affinity binding domains for CD20 and one for CD3 (Fig. 1) [81]. Ultimately long-term follow up is pending, but the lack of flattening of the tails of PFS curves call into question a curative potential for single-agent BsAbs.

## Dissecting choices among dueling immunotherapies

BsAbs therefore are efficacious and safe in patients with DLBCL. They are also effective in the notoriously hard-to-treat population of patients rel/ref after CAR-19 [82]. Yet, many patients progress on BsABs or fail to respond. BsAbs can be viewed in two ways. They appear to have less AEs and have been studied in patients with worse performance status than in the CAR-T approval studies, and hence can be considered a safe option for sicker patients and good palliative choice in later lines. Alternatively, considering their side effect profile, they may be the ideal immunotherapies for combining with other treatment modalities, an approach not yet well explored for CAR-19. Ongoing studies are assessing BsAb combinations both in early and later lines. Indeed, epcoritamab already has been assessed in frontline therapy, combined with R-CHOP in the EPCOR NHL-02 study [83]. The population included 47 patients all of whom had high-risk disease by the International Prognostic Index [84] and 11 with double/triple hit lymphoma. Complete metabolic response (CMR) was achieved in 76% and notably, response rates were similar for patients with double-hit/triple-hit DLBCL (CMR rate, 82% [9/11]). Ninety-six percent of patients with CMR maintained it at 9 months [85]. Glofitamab also was assessed in first line combinations with R-CHOP or the POLARIX regimen (Pola-R-CHP) [86]. Patients received 5 cycles of glofitamab (2.5 mg & 10 mg step-up in C2, 30 mg in C3-6) in combination with R-CHOP (Arm A) or Pola-R-CHP (Arm B). Gr ≥3 AEs were seen in 40% (Arm A) and 52% (Arm B) while Febrile neutropenia was observed in 1/25 (4%) and 6/21 (29%), respectively. No ICANS or Gr ≥3 CRS occurred. Nineteen patients (76%) demonstrated CR, 5 (20%) PR and 1 (5%) PD. PFS at 6-mo was 91% [86]. Mosunetuzumab-CHOP was assessed in 40 previously untreated DLBCL patients, giving step-up dosing of the BsAb during cycle 1, followed by fixed 30 mg on D1 of cycles 2–6 [87]. This resulted CRS in 60% (exclusively Gr1-2), a single incident of Gr 3 ICANS, and two patient deaths considered not mosunetuzumab related (pneumonia and disease progression). Two-year PFS and EFS were 65.4% and 60.4% respectively. These studies indicate feasibility of combining BsAbs with standard frontline chemoimmunotherapy but will require phase 3 randomized trials to change standards of care.

Cost is another factor that is likely to play a role when both CAR-T and BsAbs are valid clinical options. While these data are not readily available to patients and their treating physician in real time, some studies have tried to shed light on this issue. Using a hypothetical cohort of patients, axi-cel in the second line is provisionally cost-effective in selected primary r/r DLBCL patients at a willingness-to-pay (WTP) of $100,000 per quality-adjusted life-year (QALY) [88]. Another study derived similar cost of axi-cel and tisa-cel with model inputs from the ZUMA-7 and BELINDA trials [89]. This figure escalated at $271,399 per QALY when examined in third line challenging CAR-T cost-effectiveness in later lines. One approach that is likely to decrease cost to the health system is increased adoption of outpatient administration of CAR-T. This is more commonly done with liso-cel considering lower frequency of high grade CRS and ICANS although disease-specific factors also play a role [90]. Data is limited on currently approved BsAbs cost effectiveness. Blinatumomab, a BsAb used in ALL has high estimated cost of $89,000 per cycle which presents a challenge to the notion that BsAbs are cheaper than CAR-T when a treatment till progression paradigm is used [91]. Recently approved BsAbs epcoritamab and glofitamab are substantially cheaper, with an estimated cost of $37,500 and $41,176 per month of drug, respectively [92, 93]. However, BsAb treatment does also result in ancillary costs associated with AE management (e.g., in-patient administration, tocilizumab co-administration) that addon to the overall cost. Altogether, encompassing both direct treatment and ancillary healthcare infrastructure costs, it is not inconceivable that a full BsAb treatment regimen is comparable, if not more expensive in certain instances based on duration of treatment, than a single CAR-T treatment dose. With approvals now in place for both modalities, future direct cost-effectiveness comparisons are needed to clarify this vital issue.

A key question now emerging is appropriate sequencing between BsAbs and CAR-19. If BsAbs are able to improve outcomes in the frontline, CAR-T efficacy post BsAb exposure must be determined, and available data are limited. Crochet et al. assessed patients in the French DESCAR-T dataset and showed that the efficacy of CAR-T appears preserved in rel/ref B-NHL patients whose disease progressed after prior BsAb exposure [94, 95]. Extrapolation therefore hints at preserved CAR-T activity post BsAb-containing treatment, but prospective studies are needed to clearly answer this question. Of note, recent data hints and decreasing efficacy of CAR-T when delayed to third compared to second line in LBCL [96]. We are gaining insight on factors that negatively affect CAR-T fitness. ASCT and bendamustine prior to apheresis have been shown to negatively impact T cell fitness and reflect on survival [97, 98]. Another area of uncertainty is whether BsAbs will have any role like CAR-T in the second line for primary refractory or rapidly relapsing LBCL. To date, no direct comparison is available and are unlikely to be available in the near future. Thieblemont et al. conducted an indirect comparison of epcoritamab patients enrolled in EPCORE NHL-001versus axi-cel patients enrolled in ZUMA-1 with propensity score matching. Among the CAR T-adjusted matched population, ORR and CR were not statistically different in those treated with epcoritamab versus axi-cel (ORR: 73.4% vs. 74.3%, respectively; CR rate: 48.5% vs. 54.5%; *P* > 0.05). This remained true in a subgroup analysis of NHL-001 patients who were CAR-T naïve and considered CAR-T eligible [99]. A similar analysis was done for mosunetuzumab versus axi-cel and showed improved efficacy and more durable response with axi-cel [100]. Another consideration is patients’ time commitment. CAR-T, after an initial apheresis procedure, is a few days of lymphodepleting chemo before a one-time product infusion and recovery therefrom. BsAb therapy takes much longer: Glofitamab is 12 21-day cycles; epcoritamab is continued until disease progression or treatment intolerance. In patients who are CAR-T naïve in the 3^rd^ line, this is likely to play a major role in physician-patient discussions aimed at informed decisions on treatment choices. Finally, the choice of lymphodepleting agent and its impact on future immunotherapy response remains a hypothetical risk. Bendamustine was used in the BELINDA trial and was later compared with flurdarabine + cyclophosphamide showing similar efficacy and decreased toxicity [101]. Until randomized data are available to answer these question, CAR-19 remains the preferred choice in the second line for these high-risk patients. However, availability of BsAbs is likely still to impact many such patients, including those with failed apheresis or product manufacturing, in resource-limited settings, or, it could be argued, with rapidly progressing disease requiring bridging for CAR-T.

## Novel BsAb targets and combinations

Most BsAbs currently approved or under development for DLBCL target CD20. Hutchings et al reported on a novel BsAbs targeting CD19x4-1BBL (RO7227166) as a co-stimulatory molecule for glofitamab [102]. Their preclinical work showed that in the presence of a T-cell receptor signal and strictly dependent on CD19 crosslinking, RO7227166 provides a strong co-stimulation to T-cells via 41BB agonism. They showed additive benefit in a first-in-human study in rel/ref NHL that included 46 patients with DLBCL and achieved 39% CR rates with no new safety signals.

Other targets on B-cells are being actively explored and could provide the benefit of BsAb immunotherapy in a variety of clinical scenarios. CD47 is an innate-immunity checkpoint “don’t-eat-me” signal that binds macrophages and prevents phagocytosis. To counteract the immunomodulatory effect of CD47 on B cells, Hawkes et al designed a CD19xCD47 BsAb TG-1801 that blocks CD47 on CD19+ cells thus releasing macrophage anti-lymphoma activity. This showed modest 23% ORR [103]. Another product is IMM0306 that binds CD20 and CD47 on the surface of B cells with the main dose limiting toxicity being cytopenias pending further data [104]. ROR1 is a cell surface receptor expressed in wide range of hematologic and solid tumors and under exploration as a target for a CD3-ROR1 BsAb in B-NHL [105]. In a different approach, and to circumvent T cell exhaustion with CD3 binding of BsAbs, Lu et al reported on a tri-specific antibody targeting CD19, CD3 and the co-stimulatory T-cell receptor CD2 which showed safety in mammals and is being actively studied in humans with B cell malignancies [106].

Recent advances in technology have also fueled development of next-generation allogeneic CAR-T therapies, designed with the goal of circumventing the high-cost and manufacturing delays and other challenges associated with current autologous products [107]. A common strategy is to isolate T-cells from healthy non-cancer patient donors and genetically modify them to evade the immune system via genetic modifications, such as knocking-out the T-cell receptor (TCR) via a *TRAC* knock-out (KO) and/or knocking out class 1 major histocompability complex (MHC) via *β2M* KO to reduce graft-versus-host disease (GVHD) and decrease potential for rejection of the allogeneic CAR-T product by the patients’ own T-cells. Locke et al recently reported a 57.6% ORR in 33 rel/ref LBCL patients treated with an allogeneic CAR-19 that was derived from healthy T-cell donors and genetically modified with a TCR KO [108]. McGuirk and colleagues also recently showed that treatment with an allogeneic CAR-T harboring *TRAC* and MHC KO led to a 67% ORR in 32 LBCL treated patients [109]. No GVHD was observed in either study, a direct result of the allogeneic-enabling genetic engineering, and importantly no Gr3+ CRS. The promise of allogeneic CAR-T’s is in providing broader patient access driven by reduced manufacturing protocols and costs and elimination of vein-to-vein time challenges. Early clinical data suggest an improved safety profile vs. both autologous CAR-T and BsAb with no observed Gr3+ CRS, and a DoR profile that falls in between autologous CARs and BsAbs. As allogeneic CAR-Ts are further developed, consideration of their use must be accounted as it relates to sequencing and/or replacement of autologous CAR-T vs. BsAb use.

Another emerging theme is the generation of CAR-T cells directly within the patient [110]. A variety of delivery vectors, either viral and/or nanoparticle-based, can be utilized to deliver the CAR transgene directly to T-cells within the patient via intravenous infusion. A lentiviral platform for in vivo T-cell engineering, wherein viral particles were coated with anti-CD3 scFv’s to allow for T-cell targeting, showed compelling proof of concept data with a CD19 CAR-T construct in both successful biodistribution within canine models and anti-tumor activity in Nalm-6 tumor bearing mice [111]. The group also proposed a strategy to bypass lymphodepletion using a novel rapamycin-activated cytokine receptor that boosts T-cell survival and expansion in vivo, showcasing sustained B-cell aplasia in a non-human primate model through day 70 post-treatment in the absence of lymphodepletion [112]. In a different approach, Parayath and colleagues used a biodegradable polymer formulation, with mRNA for an anti-CD19 CAR encapsulated within [113]. In contrast to the lentiviral approach used by Michels et al., an mRNA-based delivery is more transient, suggesting future possibilities of repeat dosing regimens. Directly reprogramming T-cells in vivo presumably has readthrough to be even more cost effective than allogeneic CAR-T therapy given manufacturing since engineering occurs intra-patient, as opposed to external manufacturing facilities, and with patient access potentially equivalent to BsAbs. Should it be found efficacious and safe, in vivo CAR-T has the potential to disrupt the current SOC paradigm as a therapy carrying the advantages of both CAR-T and BsAb’s but with an expectation for significantly decreased costs, potentially changing the debate of CAR-T vs. BsAb’s.

## Conclusions

BsAbs represent exciting and novel therapies for patients with DLBCL. With the approvals of glofitamab and epcoritamab, patients have active options after progressing on or unable to receive CAR-19. These therapies are strong considerations for patients medically unable to wait for CAR-product manufacturing or whose socioeconomic situation or geographic location present barriers to commercial CAR-T, or potentially those who lack sufficient circulating T-cells for adequate apheresis, although this has yet to be defined in clinical practice. BsAbs achieve rapid responses and remissions ~20 months median but with no convincing signal of cure in the data available to date. The side effect profile is similar to CAR-T but occurs at a lower frequency and grade, and administration in the community is being actively implemented, which will enhance the reach of immunotherapy to a wider population. Sequencing after or before CAR-T is under active evaluation with possible roles both as additions to curative first line chemo-immunotherapy and a palliation post CAR-T failure. Emerging allogeneic and in vivo reprogramming technologies will likely add onto the clinical decision making complexity as they advance in clinical development. Future phase 3 studies are eagerly awaited to obtain full FDA approval and more clearly establish overall survival effects of BsABs. Finally, with varying targets, it’s conceivable patients may be able to receive multiple BsAbs during their cancer treatment journey, with the ultimate goal of prolonging the lives of patients with DLBCL.
